# Interorganisational Integration: Healthcare Professionals’ Perspectives on Barriers and Facilitators within the Danish Healthcare System

**DOI:** 10.5334/ijic.2449

**Published:** 2016-03-31

**Authors:** Anne Marie Lyngsø, Nina Skavlan Godtfredsen, Anne Frølich

**Affiliations:** Research Unit for Chronic Conditions, Bispebjerg University Hospital, Copenhagen, Denmark; Department of Respiratory Medicine, Hvidovre University Hospital, Hvidovre, Denmark

**Keywords:** interorganisational, integration, barriers, facilitators, Denmark

## Abstract

**Introduction:**

Despite many initiatives to improve coordination of patient pathways and intersectoral cooperation, Danish health care is still fragmented, lacking intra- and interorganisational integration. This study explores barriers to and facilitators of interorganisational integration as perceived by healthcare professionals caring for patients with chronic obstructive pulmonary disease within the Danish healthcare system.

**Methods:**

Seven focus groups were conducted in January through July 2014 with 21 informants from general practice, local healthcare centres and a pulmonary department at a university hospital in the Capital Region of Denmark.

**Results and discussion:**

Our results can be grouped into five influencing areas for interorganisational integration: communication/information transfer, committed leadership, patient engagement, the role and competencies of the general practitioner and organisational culture. Proposed solutions to barriers in each area hold the potential to improve care integration as experienced by individuals responsible for supporting and facilitating it. Barriers and facilitators to integrating care relate to clinical, professional, functional and normative integration. Especially, clinical, functional and normative integration seems fundamental to developing integrated care in practice from the perspective of healthcare professionals.

## Introduction

The Danish healthcare system, like most Western health systems, is facing an increasing proportion of individuals with one or more chronic conditions. These individuals often have multiple care demands and require complex and continuous services spanning professions, sectors and political levels [1,2,3]. A 2012 review reports that the Danish health system generally provides high-quality care and that patient satisfaction is high. However, despite many initiatives to improve the coordination of patient pathways and intersectoral cooperation, Danish healthcare is still fragmented and lacks intra- and interorganisational integration [4]. Common to most Western health systems is an increasing differentiation of roles, tasks and responsibilities resulting from efforts to decentralise, specialise and professionalise healthcare systems. Although these efforts have the desired impact in terms of clinical and organisational differentiation, they also engender fragmented care [5,6].

A fragmented system can be defined as one lacking the integration required to achieve unity of effort [7]. Each part of the system tends to focus on internal tasks and resources, overlooking the system as a whole. Internationally, a number of studies have identified some general determinants for integrating health care [8,9,10,11]. Reviews of integrated care initiatives and measurement instruments find that their success and factors that work as barriers or facilitators in terms of integrating care depend substantially on context [12,13]. Therefore, national solutions cannot necessarily be adapted in new settings without paying close attention to clinical, geographic, financial and policy contexts.

### Organisation of the Danish healthcare system

Despite a number of reforms and policy initiatives that have gradually centralised the system, Danish health care is still characterised as fairly decentralised with responsibility for primary and secondary care located at local levels. The health system is organised at three administrative levels: the state, five regions and 98 municipalities. The state regulates, supervises and finances health care and is increasingly taking responsibility for planning activities such as quality monitoring and the distribution of medical specialties in hospitals. Regions are responsible for hospitals and self-employed healthcare professionals such as general practitioners, specialists, dentists, physiotherapists, chiropractors and pharmacists. Municipalities are responsible for disease prevention, health promotion and rehabilitation for people with chronic conditions. General practitioners act as gatekeepers, referring patients to hospital and specialist treatment, and are compensated by the regions on a combined capitation and fee-for-service basis. Most secondary and tertiary care takes place in general hospitals owned and run by the regions. Hospitals include inpatient care, outpatient clinics and 24-hour emergency wards [4,14].

### Strategies to improve the integration of care in the Danish healthcare system

The integration of healthcare services has been on the health policy agenda in Denmark since the 1980s. In 2007, a major structural reform of the Danish public sector took place, the culmination of a decade-long series of interventions that attempted to strengthen coordination and centralise control. The reform reduced the number of administrative units at regional and municipal levels, and an explicit goal of the reform was the integration of health services. An important element was the introduction of mandatory healthcare agreements between the regions and municipalities that contain a set of common goals and mutual commitments and collaboration. Instituted at the start of the regional and municipal election cycle every 4 years, agreements cover six areas: hospital admission and discharge processes, rehabilitation, medical advice and assistance, prevention and health promotion, mental health and follow-up after adverse events. The effect on coordination of the 2007 reforms has been evaluated by Rudkjøbing et al., who found that healthcare agreements are considered a useful tool for strengthening coordination between the regions and the municipalities – and that challenges persist [15,16]. Other recent initiatives to improve service integration include financial incentives to coordinate care for chronically ill patients in general practice, development of care pathways for 32 types of cancer that specify predefined courses of action and chronic disease pathway programmes for 10 conditions that consist of standardised descriptions of interdisciplinary, cross-sectoral and evidence-based care and cooperation and coordination between actors. Furthermore, full implementation of a shared medication record system across settings is stated to take place in mid-2016 in two Regions of Denmark. However, the many reforms and initiatives to integrate services at the administrative level may not fundamentally alter how physicians and other frontline staff collaborate. Thus, as Rudkjøbing et al. [15] note, the work that comes after signing healthcare agreements and preparing and agreeing on other initiatives actually produces coordination. Consequently, the aim of this study is to describe what healthcare professionals and managers within the Danish healthcare system perceive as barriers to and facilitators of interorganisational integration and what they think is needed to increase interorganisational integration.

## Theoretical framework

Integrated care is a key strategy in reforming health systems around the world. However, ‘integrated care’ is a nested concept with a lot of embedded meanings. The lack of conceptual clarity is a major barrier to promoting integrated care, greatly hampering systematic understanding, successful real-world application and meaningful evaluation [2,5]. Many professionals engaged in the process of understanding integrated care have produced various definitions for the concept, and analysts have distinguished different dimensions of integration. The literature has proposed six different dimensions of integration, each of which is enabled through a range of integrative processes: *functional integration* (the degree to which back-office and key support functions such as financial management, human resources, strategic planning, information management and quality improvement are coordinated across all operating units); *organisational integration* (the extent to which services are produced and delivered in a linked fashion between healthcare institutions); *professional integration* (intra- and interorganisational professional relationships between healthcare providers); *service or clinical integration* (the extent to which patient care services are coordinated across various professional, institutional and sectoral boundaries in a system); *normative integration* (shared mission, vision, values and organisational/professional culture); and *systemic integration* (coherence of rules, policies and incentives at all organisational levels). Valentijn and colleagues recently created a unifying conceptual framework that organises key features for achieving integrated service delivery into the six dimensions of integration. The work is an essential building block in the process of understanding the complex phenomenon of integrated care and suggests that integration must be pursued at different levels within a system to facilitate the continuous, comprehensive and coordinated delivery of services to individuals and populations. The conceptualisation shows how the different dimensions of integration play complementary roles on the micro- (clinical integration), meso- (professional and organisational integration) and macrolevel (system integration) to deliver comprehensive services. Additionally, it underlines how functional integration and normative integration span the micro-, meso- and macrolevel and ensure connectivity between the levels [5,17,18,19,20,21].

The theoretical framework is helpful in understanding how the perspectives of healthcare professionals and managers relate to the different dimensions of integration and hence which dimensions to prioritise when trying to improve care integration. The relation between our results and the theoretical framework will be discussed in the Discussion section.

## Methods

### Data collection

A qualitative research approach was used to explore the experiences and viewpoints of healthcare professionals and managers caring for patients with chronic obstructive pulmonary disease in the Danish health care system. Seven semi-structured focus groups were conducted with 21 informants (17 healthcare professionals and four managers) from general practice, two local healthcare centres and a pulmonary department at a university hospital in the Capital Region of Denmark (Table 1). The aim of the focus group interviews was to explore factors that healthcare professionals and managers within the Danish healthcare system perceived as barriers to and facilitators of integrating care and what they thought was needed to increase interorganisational integration.

**Table 1:** Characteristics of informants (*n* = 21).

	GROUP						

	**1** ***n* = 2**	**2** ***n* = 5**	**3** ***n* = 2**	**4** ***n* = 4**	**5** ***n* = 4**	**6** ***n* = 2**	**7** ***n* = 2**

Profession							
General practitioner						2	1
Nurse (General Practice)							1
Chief Physician (Pulmonary Dept.)	1						
Head Nurse (Pulmonary Dept.)	1						
Physician (Pulmonary Dept.)		3					
Nurse (Pulmonary Dept.)		2					
Manager (Healthcare Centre)			2				
Nurse (Healthcare Centre)				2	2		
Physiotherapist (Healthcare Centre)				2	2		

Data collection took place in January–July 2014 at informants’ workplaces. Each interview lasted 90–120 minutes and was conducted by the first author. A semi-structured interview guide included open-ended questions that were based in part on a systematic review of organisational elements important to the process of creating integrated care [12]. All interviews were audio-recorded, transcribed by a research assistant who observed each interview and coded using NVivo 10 software.

### Recruitment

The managers of two local healthcare centres and the chief physician and head nurse of the pulmonary department at the university hospital were invited to participate. They subsequently recruited other healthcare professionals based on the inclusion criteria of nurses, physiotherapists and physicians who had been working at the centres or the hospital for at least 6 months and treated people with chronic obstructive pulmonary disease. General practitioners were contacted by mail and received a follow-up call with an invitation to participate in the study.

### Analysis

The first author and a research assistant coded the interview data. They individually coded a data sample and reviewed the results, generating a code manual used for subsequent coding. Throughout the analysis, they discussed and resolved any uncertainties or differences in coding, adding additional codes by mutual agreement. Themes identified in the analysis emerged from the data, rather than being based on predetermined categories. The focus of the analysis was on understanding informants’ experiences with barriers to and facilitators of integrated care and their opinions about processes that could help increase interorganisational integration.

### Ethical approval

The study was approved by the Danish Data Protection Agency; 2007-58-0015, BBH-2013-037, I-Suite: 02488. All informants gave written consent to participate in the interviews.

## Results

Five themes emerged from interviews: (1) communication/information transfer; (2) committed leadership; (3) patient engagement; (4) the role and competencies of the general practitioner; and (5) organisational culture. As articulated by informants, barriers and facilitators were often two sides of the same coin (e.g. good leadership/poor leadership). Consequently, the themes discussed below include barriers and facilitators. Table 2 summarises barriers experienced by the informants and their thoughts about facilitators or solutions to existing challenges.

**Table 2:** Barriers and facilitators to integrating care.

Area	Barriers	Facilitators/solutions

Communication/information transfer		
*Information technology systems*	No integrated information system to facilitate transfer of information across settings	An electronic system accessible across settings making it possible to search for relevant patient information like referrals, discharge letters, test results and short annual resumes about patients and their treatment
*The diagnostic phase*	Inadequate referrals due to lack of information about the medical regimen, smoking status and old or missing test results	Improve quality of care in general practice through a focus on early detection and interpretation of test results
		Clear referral procedures
		Knowledge-sharing meetings with representatives from each setting discussing a sample of patient cases to address incentives, barriers, strengths and weaknesses and opportunities to provide high-quality and well-integrated patient pathways
*The phase between regular visits at the outpatient clinic for the very severe patients*	No opportunities for patients and general practitioners to get advice or help between regular visits at the outpatient clinic	A 24-hour nurse-led chronic obstructive pulmonary disease-specific hotline service at the hospital available for both patients and general practitioners
		A case manager with specific training and expertise in caring for patients with chronic obstructive pulmonary disease, who contacts patients directly to ensure that they attend appointments and adhere to their medications. In addition the case manager would facilitate access to care services in other departments of the hospital and in the municipality and coordinate aspects of social care services, such as home care
*The hospital discharge phase*	Discharge letters from the hospital are often inadequate due to lack of information about changes in the medication regimen and a missing rationale for the changes	Clear discharge procedures and a higher priority to producing discharge letters in the hospital
	Discharge letters also miss an adequate description of the future care plan, including the patients’ goals and preferences	Knowledge-sharing meetings with representatives from each setting discussing a sample of patient cases to address incentives, barriers, strengths and weaknesses and opportunities to provide high-quality and well-integrated patient pathways
Committed leadership	Leaders who are not committed and do not communicate clearly about the importance of integrated care	Managers consistently sharing a vision of integration with their employees
	Front line staff unwilling to take responsibility	Managers acknowledging tasks related to interorganisational integration and prioritising and allocating time for completing them
		Informal network meetings between managers from each setting
Patient engagement	Professionals planning and communicating in a triangle around the patient	Shared decision-making
		The use of patients’ own resources
		Patient activation and responsibility
The role and competencies of general practitioners	At the healthcare centres and the hospital, managers and clinicians received a remarkably small number of referrals from general practice; very often inadequate because of missing information or dated test results	Improve quality of care in general practice; focus on early detection and interpretation of test results
		Clear referral procedures
		Clinical guidelines with clear directions on the management of comorbidities
		Knowledge-sharing meetings with representatives from each setting discussing a sample of patient cases to address incentives, barriers, strengths and weaknesses and opportunities to provide high-quality and well-integrated patient pathways
Organisational culture	Differing perspectives, cultures and working conditions in different sectors created a great need for understanding the concerns and needs of others	Knowledge-sharing meetings with representatives from each setting discussing a sample of patient cases to address incentives, barriers, strengths and weaknesses and opportunities to provide high-quality and well-integrated patient pathways
		Spending time at others’ work places

### Communication/information transfer

Effective communication and information transfer across the interface between primary and secondary care was seen as the factor most vital to integrated care. Informants raised issues related to both information technology systems and the content of transferred information. With respect to the latter, three phases of care were identified as areas for improvement: (1) the diagnostic phase, in which informants perceived appropriate referrals from general practice as vital; (2) the phase between regular outpatient visits, in which a telephone hotline service and a case manager were needed; and (3) hospital discharge, after which patients returned to general practice.

#### Information technology systems

When discussing information technology systems, informants agreed that a major barrier to effective care transitions was a lack of a shared system to facilitate transfer of information across settings. The manager of a local healthcare centre said:

An incentive for a closer, an ever closer cooperation with the hospital and general practice would be communication channels. If it is easy to get in contact, if you do not have to be in a waiting position, if you do not have to wait for days to get an answer (…) that would be very facilitating and I think that it would improve the intersectoral collaboration.

At the time of the study, information technology solutions were available in each setting. Transfer of information mainly occurred by fax, e-mail, electronic data interchange or correspondence messages (an electronic tool that can be used instead of emails and phone calls to send short messages or queries about a patient across settings). Informants reported that none of these solutions were adequate, although they varied considerably in the use of electronic data interchange and correspondence messages. The latter were available in each setting but used only to transfer information between general practice and the municipality; informants were unaware they could also be used across the primary/secondary care interface. Informants expressed a common desire for a standardised electronic system that was accessible across settings and could be searched for relevant patient information, e.g. referrals, discharge letters, test results and short annual summaries about patients and their treatment from general practice and the outpatient clinic.

#### The diagnostic phase

With respect to the content of transferred information, informants perceived referral of patients from general practice to rehabilitation at local healthcare centres and the hospital as a major area for improvement. Informants from the health-care centres and hospital noted the low number of patients referred to the rehabilitation programmes at their organisations. Informants in general practice expressed awareness of the potential to refer more patients and the need for a more systematic approach. General practitioners and their nurses attributed the low number of referrals to the fact that the rehabilitation programmes had existed for a relatively long time without conducting promotional activities. A local health centre tried to address this issue by visiting all local general practitioners to remind them about programmes and referral procedures. Informants from local health centres and the hospital shared experiences in which referral procedures were only followed by some general practitioners, a gap that often resulted in a lack of information about the medical regimen, smoking status and current test results. This led, in turn, to wasted time, repeated tests and uncertainty about the primary purpose of the referral for both receiving clinician and patient. In addition, uncertainty about the purpose of referral was exacerbated when general practitioners failed to communicate a precise diagnosis and disease stage in the referral or when patients were not adequately prepared for care in the next setting. Inadequate information often contributed to anxiety and dissatisfaction for patients and professionals.

#### The phase between regular visits at the outpatient clinic for patients with severe illness

The second area for improvement in information transfer was the interval between regular outpatient clinic visits. At this stage, informants perceived two factors as particularly important to creating integrated care: (1) the availability on demand of advice from a nurse at the hospital with in-depth knowledge of chronic obstructive pulmonary disease; and (2) the availability of a case manager. Managers and healthcare professionals at the hospital perceived a need among patients with severe disease for a 24-hour nurse-led telephone hotline service. Patients with more severe disease often did not keep appointments for regular outpatient clinic visits because of acute hospital admissions; participants shared the perception that the hotline service could reduce such hospitalisations. Participants from general practice also found this service crucial in their daily practice and to integrated care in general.

With respect to the availability of a case manager, professionals at the hospital reported that this role could promote cooperation with general practice and the municipality and have an important impact on the integration of services across sectors. The case manager would be a healthcare professional with specific training and expertise in caring for patients with chronic obstructive pulmonary disease, contacting them directly to ensure that they attended appointments and adhered to their medications. In addition the case manager would facilitate access to care services in other departments of the hospital and in the municipality and coordinate aspects of social care services, such as home care.

#### The hospital discharge phase

A final area for improved communication was the discharge phase. It was a common experience among informants in general practice that discharge letters from the hospital were the most important tool to ensure a safe and integrated transfer of patients, but they were often inadequate. A general practitioner said:

If I were to say something about the intersectoral issue, then the discharge letter they (the specialists) write, it is actually the most valuable thing to the patient, and I have the impression that it is not the best qualified who author it and is responsible for it. Perhaps the signature, but I think they just quickly read through what another junior writes (…) the transition from one sector to another should have a higher priority. It is very important and it is often neglected. They may do something good in there, but if I do not get a good and understandable message back, they may quickly go back again.

Informants expressed a need to place a higher priority on producing discharge letters in hospital, highlighting two types of information. The first was a clear description of and rationale for changes in the medication regimen at discharge. The second was an adequate description of future care plans, including patient goals and preferences. Descriptions of future care plans were desired both from the local healthcare centres and from general practice.

### Committed leadership

The importance of leadership was mainly addressed by healthcare professionals from the hospital and local health-care centres. Managers and professionals in both organisations agreed that committed leadership with clear communication processes and front line staff members who are willing to take responsibility for communication are crucial when bringing different cultures together. The manager of one of the local healthcare centres explained:

It is simply a task for the chief physician or the head nurse. It is simply another responsibility of theirs, to ensure that the cooperation is also running. Before we get there – and it’s got nothing to do with the head of the hospital – it’s about the chief physician and the head nurse.

Accordingly, informants explained that it was important for managers to consistently share a vision of integration with employees. Furthermore, informants at the local healthcare centre found it important that managers both acknowledged tasks involved with interorganisational integration and supported allocating time to complete them. They perceived it as important that managers arranged social events with opportunities to interact with healthcare professionals from other organisations because they experienced personal connections as facilitating collaboration. Managers at the hospital and local healthcare centres noted that it could be very useful to conduct regular informal network meetings with managers from each setting and stressed the importance of establishing this in the near future.

### Patient engagement

Informants generally viewed patient engagement as a very important factor in relation to integrated care. In particular, informants from the local healthcare centres and general practice articulated how shared decision-making, the use of patients’ own resources and patient activation and responsibility were intended to have a crucial impact on health outcomes, patient experiences and the level of integration between organisations. In relation to shared decision-making, managers and healthcare professionals at local healthcare centres described how they always sat down with patients to discuss their condition, treatment options and benefits, and preferences and motivation. They often considered it important to deviate from the plan as provided in the referral to enable patients to be the managers of their treatment plans. As one informant stated, their task as healthcare professionals was to guide individual patients in a way that made it possible for them to find their own care path. Managers of local healthcare centres highlighted the importance of paying attention to and making use of patients’ resources:

Sometimes we have a tendency to underestimate their resources. Even the weakest, those of whom we have thought ‘oh, well they are the most disadvantaged people’, they have a lot of resources (…) But I also think, that we could do more than we already do in this area to support the individual. We could give them the opportunity to have the overview themselves.

In general practice, patients with chronic obstructive pulmonary disease were viewed as very vulnerable and with limited ability to care for themselves, particularly with respect to medications. General practitioners noted that they had to pay special attention to patients’ medical adherence and ensure that patients knew about their drugs and their use. Poor adherence was viewed as a risk factor for emergency hospitalisation, thus affecting the system as a whole.

Informants from all three organisations noted that it was crucial to place the patient in the centre of the treatment instead of having professionals from general practice, the municipality and the hospital planning and communicating in a triangle around the patient. There was a strong emphasis on how to support patients to encourage them to take responsibility and coordinate their care. Words like ‘patient activation’ and ‘the active patient’ were used to describe patients with the skills, ability and willingness to manage their health and care. Active involvement of patients was intended to have a crucial impact on the overall integration between organisations.

### The role and competencies of general practitioners

There was a general agreement among informants that general practice played a crucial role in integrating and coordinating care for patients with chronic conditions and multiple morbidities. General practitioners’ role as gate keepers to more specialised treatment was highlighted as particularly important, and they were seen as central to facilitating the smooth transition of patients across organisational boundaries.

Informants from the hospital and local healthcare centres noted that coordination and collaboration with general practice was complicated by general practitioners’ diversity in terms of knowledge, specialty, patient clientele and interests. Particularly with regard to the early detection of disease, managers and clinicians at the local healthcare centres and at the hospital found varied levels of competence among general practitioners. They viewed some as highly skilled and using a very systematic approach, whereas others did not know how to perform a spirometry test and interpret the results. Even general practitioners who felt competent at diagnosing and treating patients with chronic obstructive pulmonary disease noted that they could be more proactive and transfer more patients to rehabilitation at the local healthcare centres or hospital. This represents a paradox, in that a core role in general practice is to provide continuity of care to patients and act in a coordinating role through referrals to other services, yet some general practitioners seemed to play a tangential rather than central role in the care process. In terms of the number of referrals, general practitioners stated that clinical guidelines for managing single chronic conditions often failed to offer clear direction for managing comorbidities; patients who should be referred to rehabilitation according to guidelines were often not referred because of comorbidities or patient preferences. All general practitioners found that flexibility in regard to guidelines was important to enabling them to make the best use of their skills to tailor care to the individual needs of patients.

### Organisational culture

#### Different perspectives and goals and the feeling of shared responsibility

Informants identified differing organisational cultures as a main barrier to developing integrated care pathways. Care in hospitals was focused on acute and episodic care, which contrasted sharply with the holistic and long-term perspective in general practice and local healthcare centres. A hospital manager noted:

We treat chronic obstructive pulmonary disease patients at different stages, life phases and disease stages … we probably see them in here, where we are less likely to see them as the person they are, the human being they are and the context they are in, whereas in the primary sector, they probably see them more as the individual, Mr. Jensen, and have less focus on the impact of the disease.

General practitioners agreed with this perspective and found that it made shared goal setting very difficult. Whereas they felt they had a thorough knowledge of their patient’s everyday life and made an effort to incorporate this into their approach, they did not see this as the case among their colleagues at the hospital. At local healthcare centres, managers and healthcare professionals experienced the care perspective at the hospital as different in the rehabilitation unit than in the general inpatient wards and outpatient clinic. They believed that there was a more holistic and long-term perspective within the rehabilitation unit, compared to other parts of the hospital that were dominated by an acute care mindset. The focus on survival and acute care was viewed as a consequence of both time pressure and old habits that left no room for innovation and greater patient involvement.

Managers of the pulmonary department at the hospital added the perspective that seeing patients at different disease stages indirectly impacted goal setting. In theory, they believed they had the same vision about treatment as in primary care; however, in practice, they knew that things were different, largely due to time constraints. Participants’ differing perspectives also seemed to have an impact on whether or not treatment was seen as a shared responsibility across settings. At local healthcare centres, treatment was experienced as a shared responsibility when communication with general practice and the hospital was successful and their efforts were recognised by other professionals. Informants at local healthcare centres felt motivated when intersectoral cooperation went smoothly and stressed when it did not. They found it important that everyone involved in the treatment shared a more consistent and enduring feeling of responsibility.

#### Respect and trust building

Informants shared a common understanding that respect and trust are important to successful collaboration and that time is required to build and sustain these qualities. In all three organisations, managers and healthcare professionals noted that they had made progress in relation to building mutual respect and trust, although some professionals described it as an ongoing process. Managers from both organisations noted that rapid changes in staff affected the building of personal connections and trust and that interorganisational cooperation was very vulnerable if it relied on just a few employees.

Furthermore, healthcare professionals at local healthcare centres sometimes perceived that hospital doctors mistrusted their skills and conveyed an attitude that their work was less important than acute management of inpatients. Informants at the local healthcare centre generally felt that healthcare professionals at the hospital were helpful about answering questions but did not function collaboratively to a satisfactory degree. One of the informants said:

I think that our role is that we have to be insanely extroverted because it is uphill in relation to the hospital. We have a … well … we have a different status and I simply think that we have to be open … I think a lot can be done to make the cooperation smoother, because you can go in opposite directions and then say, well it is extremely annoying that it is like this. I actually think so, but I do not think that we get anywhere with that, so I am now … I am extra kind to the nurse at the hospital … I act that way … because it … I get more answers by doing it. It eases my workflow because I have … I have no … I do not have any doctor … sometimes I need professional knowledge different from that of nurses. I need to have it from somewhere and I want it from real life (…) I think that the initiative has to come from our side. I wish we could allocate more resources towards it.

Informants in general practice noted that things had changed over the last 20 years, including a higher level of trust and respect between themselves and healthcare professionals at the hospitals. However, a continuing problem they noted was that hospital specialists seemed unaware of the conditions in general practice, seeming to focus on the treatment of the disease without taking into consideration the daily life of the patient and the existence of comorbidities.

#### Building relationships and agreements

Differing perspectives, cultures and working conditions across different sectors created a great need for understanding the concerns and needs of others. Local healthcare centre managers saw it as very problematic that only a few general practitioners and hospital clinicians knew about their existence and available services. Participants identified strategies to help bring the sectors together, enabling them to understand each other’s roles and engender a perspective that their work was complementary to that of others. They mentioned two main strategies. The first was knowledge-sharing meetings at which representatives from each setting discussed a sample of patient cases to address incentives, barriers, strengths, weaknesses and opportunities to provide high-quality and well-integrated patient pathways. The second strategy was allocated time to shadow colleagues working in other sectors. All informants shared the perspective that physical meetings created a form of binding relationship and a culture of mutual respect for integrated care thinking and task distribution. Knowledge-sharing meetings had been taking place for some years but were irregular at the time of the study, and general practitioners no longer participated due to time constraints. All informants agreed that participation from all sectors was crucial to ensuring a useful outcome. In terms of spending time at others’ work places, informants from the local healthcare centres and the hospital had primarily practised this, finding it a very fruitful way of understanding others’ tasks and resources. One informant from the local healthcare centre described it this way:

I also believe that it gives you another form of respect for others’ work. Like if you follow your secretary at work for a day, you also get to see, well this is why she wants to do it this way. Then she may get another feeling of responsibility and well it may mean a lot to them that they get an adequate referral, because then I can get an adequate discharge letter which in the end will make it a lot easier for me. You get an understanding of others work, you know their face. Suddenly the health-care centre is not just a thing, but Bente and Lars.

## Discussion

This study identified five main areas as crucial to integrating care at the interface between primary and secondary care.

### Comparison with other studies on interorganisational integration

Our results are similar to those of studies highlighting the importance of integrated information systems and clear referral and discharge procedures [22,23] and studies identifying the crucial impact of establishing a common organisational culture fostered by knowledge-sharing meetings, committed leadership and the appointment of a case manager [7,9]. The impact of an interface that reflects the patient perspective is also assessed elsewhere in the literature [20,24].

In relation to the theoretical framework, our findings show that barriers and facilitators mainly relate to clinical, professional, functional and normative integration. Of these four dimensions, clinical, functional and normative integration seemed most important to informants. According to Valentijn et al., functional integration and normative integration are enablers for achieving integrated care and support and link clinical (microlevel), professional and organisational integration (mesolevel) dimensions within a system (macrolevel) [18,19]. In terms of functional integration, participants highlighted the importance of shared information systems that enhance communication capacity and information flow across sectors; in terms of normative integration, they mentioned the crucial impact of shared goals and an integrative culture. Furthermore, in relation to clinical integration, the informants mentioned a range of areas in need of improvement: referrals and discharge letters, the need for advise between regular visits at the outpatient clinic, patient engagement and the competencies of general practitioners in relation to their central role in the care process. The weight on barriers connected to clinical integration indirectly states that we should start by integrating from the bottom up but also that it is important to use a range of tools to support integrated care. From the perspective of healthcare professionals and managers, future research and interventions should start with focusing on how best to overcome barriers related to clinical, functional and normative integration.

### Comparison with the patient perspective

Successfully integrating care requires ongoing patient involvement to ensure that user needs and expectations are addressed. Consequently, research and evaluations related to integrated care should include patient and family experiences [20]. A 2014 qualitative study evaluated experiences of integrated care across care settings among patients with chronic obstructive pulmonary disease and their relatives. Patients were asked about where they experienced lack of integration in the care process and their suggestions for optimising care processes [25]. Several barriers and facilitators appear in the findings of that study and here. The implementation of an integrated information system accessible to all health professionals was seen as the most central factor to integrating care at the primary/ secondary care interface. Other shared facilitators were a nurse with in-depth disease knowledge to guide patients and relatives in coping with symptom exacerbations, a case manager to coordinate healthcare and social services across sectors, the active involvement of patients in their care and proper follow-up after hospital discharge. Finally, both studies identified a need to improve the quality of care for chronic obstructive pulmonary disease in general practice.

### Limitations and strengths of the study

This study was conducted among healthcare professionals and managers working with patients with chronic obstructive pulmonary disease within a selected geographical area in Denmark; the results apply to other patient groups or settings to an unknown extent. In addition, the findings represent the perspectives of healthcare professionals and managers and cannot be generalised to other actors (e.g. patients, policymakers) involved in integrating care. However, our comparison with previous research demonstrates similarities between perspectives of patients and healthcare professionals and managers.

The ideal size of focus groups is four to eight people, a size that can help people explore and clarify their views in ways that occur less easily in one-to-one interviews [26]. However, four of our focus groups consisted of two people. It was very difficult to find a time for general practitioner focus groups that suited more than a few practitioners. The study would have been strengthened by the inclusion of more general practitioners, so each group could have consisted of at least four. In contrast, the composition of the other groups was considered carefully. We aimed for homogeneity within groups to avoid the possible effect of a hierarchy, e.g. employees feeling inhibited about sharing their experiences because of the presence of their managers. The decision to separate employees from managers also resulted in two small focus groups.

Although it limits the generalisability of our results, the qualitative nature of the study is an important strength because it offers a rich insight into elements and processes crucial to integrating care. A pressing need exists to develop measures of the degree of integration within the Danish healthcare system and in health systems in general. Qualitative studies are an important tool to identify essential elements for integration and one of the first steps in accomplishing this goal.

## Conclusion

Barriers and facilitators related to integrating care from the perspectives of healthcare professionals and managers can be grouped into five areas that are consistent with previous research in the area. Proposed solutions to barriers in each area hold the potential to improve care integration as experienced by the individuals responsible for providing it. Barriers and facilitators to integrating care relates to clinical, professional, functional and normative integration. Especially, clinical, functional and normative integration seems fundamental to developing integrated care in practice from the perspective of healthcare professionals.

## Competing interests

The authors declare that they have no competing interests.
